# High risk of early recurrent stroke in patients with near-occlusion with full collapse of the internal carotid artery

**DOI:** 10.1007/s00234-024-03283-5

**Published:** 2024-01-09

**Authors:** Alexander Henze, Allan J. Fox, Elias Johansson

**Affiliations:** 1 Institution of Radiation Sciences, Department of Diagnostic Radiology, Umeå University, Umeå, Sweden; 2 Sunnybrook Health Science Center, Department of Medical Imaging, University of Toronto, Toronto, ON Canada; 3 Institution of Clinical Science, Department of Neurosciences, Umeå University, Umeå, Sweden; 4 Wallenberg Center of Molecular Medicine, Umeå University, Umeå, Sweden; 5 Institute of Neuroscience and Physiology, Department of Clinical Neuroscience, Sahlgrenska Academy, Gothenburg, Sweden

**Keywords:** Carotid stenosis, Near-occlusion, Full collapse, Risk, Stroke

## Abstract

We aimed to validate the prognostic ability and assess interrater reliability of a recently suggested measurement-based definition of near-occlusion with full collapse (distal ICA diameter ≤ 2.0 mm and/or ICA ratio ≤ 0.42). 118 consecutive patients with symptomatic near-occlusion were prospectively included and assessed on computed tomography angiography by 2 blinded observers, 26 (22%) had full collapse. At 2 days after presenting event, the risk of preoperative stroke was 3% for without full collapse and 16% for with full collapse (p = 0.01). At 28 days, this risk was 16% for without full collapse and 22% for with full collapse (p = 0.22). Interrater reliability was perfect (kappa 1.0). Thus, near-occlusion with full collapse should be defined as distal ICA ≤ 2.0 mm and/or ICA ratio ≤ 0.42 in order to detect cases with very high risk of early stroke recurrence.

**Supplementary Information:**

The online version contains supplementary material available at 10.1007/s00234-024-03283-5.

## Introduction

**Fig. 1:**
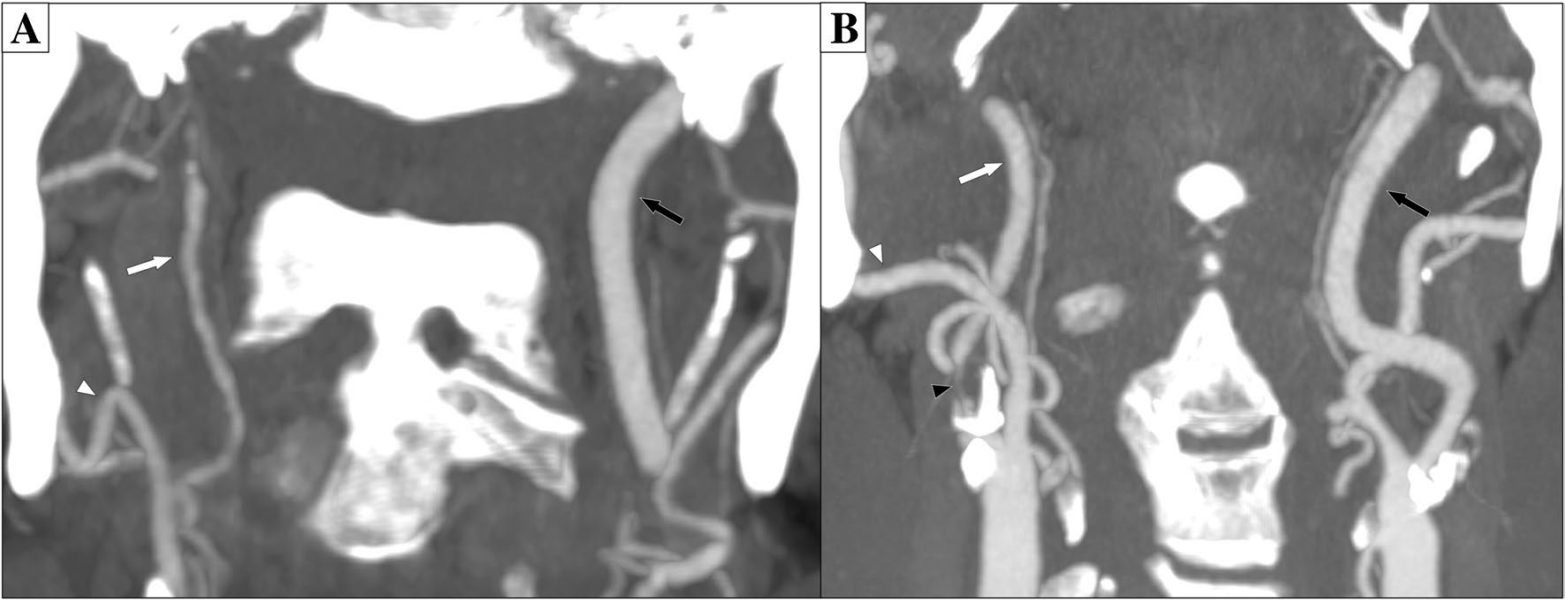
Two cases of right-sided near-occlusion on CTA. **A** Near-occlusion with full collapse. After severe stenosis (not in plane), distal ICA is threadlike (white arrow, 1.0 mm), clearly smaller than contralateral ICA (black arrow, 4.3 mm, ICA ratio 0.23) and smaller than ipsilateral ECA (white arrowhead, 2.1 mm). Full collapse by measurements and by appearance for both observers. **B** Near-occlusion without full collapse. After severe stenosis (black arrowhead), distal ICA is normal-appearing (white arrow, 2.4 mm), but smaller than contralateral ICA (black arrow, 4.3 mm, ICA ratio 0.56) and like ipsilateral ECA (white arrowhead, 3.0 mm)

Carotid stenoses are often graded with percent by comparing stenosis diameter with the diameter of the distal internal carotid artery (ICA). But severe stenoses may cause a diameter reduction in the distal ICA (“collapse”), such stenoses are to be graded as near-occlusions instead of a percent degree [1–3]. Near-occlusions can be subdivided into those with severe distal ICA collapse (full collapse, Fig. 1A) and those where the ICA is collapsed but still normal-appearing (without full collapse, Fig. 1B). Recent guidelines recommended that symptomatic near-occlusion should only undergo carotid endarterectomy (CEA) or stenting after multidisciplinary review in cases with repeated symptoms despite best medical therapy, but further studies were urged [4]. Near-occlusion with full collapse has been reported to cause high risk of early recurrent stroke [5, 6], but not unanimously [7]. Traditionally, the separation of with and without full collapse was done by assessing the appearance of the distal ICA without a clear threshold. As future management might be different between with and without full collapse, a prognosis-derived definition of full collapse on computed tomography angiography (CTA; distal ICA diameter ≤ 2.0 mm and/or ICA ratio ≤ 0.42) was recently suggested to define full collapse [8]. This threshold has not been validated and has unknown interrater reliability. The aims of this study were to assess the risk of recurrent stroke in symptomatic near-occlusion with full collapse defined by this threshold and assess the interrater reliability of this threshold.

## Methods

Prospective observational cohort study. We included consecutive patients at @blined site name@, a tertiary stroke center, between February 2018-May 2022 with a 6-month break in 2020 due to Covid19. Inclusion criteria were symptomatic carotid near-occlusion and informed consent. Exclusion criteria were clearly ineligible for carotid intervention (severe co-morbidity, very advanced age) or CTA (contrast allergy, severe kidney failure). If not done in clinic, CTA was performed as part of the study. The study was approved by the regional ethics board in Umeå, Sweden.

All events were assessed by EJ, blinded to CTA findings. Recurrent stroke was defined as in a previous study [6]: Either conventional stroke or retinal artery occlusion, only counting ipsilateral ischemic events occurring after presenting event but before revascularization. Single anti-platelet therapy (usually aspirin) was preferred unless another indication for anti-coagulation existed (such as atrial fibrillation).

Near-occlusion was diagnosed when a stenosis caused the distal ICA to reduce in size. This was assessed on CTA by systematic feature interpretation acknowledging other causes for small distal ICA [6, 9]. A conservative approach was used, only diagnosing near-occlusion when sufficiently clear. EJ assessed all exams, AJF assessed 62 exams and AH 56 exams, disagreements about near-occlusion status were resolved by consensus.

EJ measured all cases by first assessing and then measuring (expert approach) like in previous studies [6, 8], i.e. choosing a representative segment well beyond the stenosis (usually at C2 vertebrae level) and measuring by placing caliper in the middle of the fuzzy edge. AH measured the distal ICAs at mid-C2 vertebrae level using a semiautomatic centerline approach (semi-blind measurement), similar to routine practice. For both observers, measurements were taken before consensus discussion about near-occlusion status, one measurement was taken per ICA and tiny artery segments in stenosis and distal ICA where contrast opacity was reduced (presumed partial volume effect) were assigned 0.5 mm diameter when visible and 0.2 mm when not visible (but flow was obviously existent). Full collapse was defined by measurement (≤ 2.0 mm distal ICA diameter and/or ≤ 0.42 ICA ratio) where ICA ratio is calculated as ipsilateral distal ICA / contralateral distal ICA [8].

## Statistics

Where appropriate, we used mean with standard deviations (SD), median with inter quartile range (IQR), 95% confidence intervals (95%CI), 2-sided χ^2^-test, t-test, Mann–Whitney test and log rank test. Interrater reliability of full collapse by measurements was assessed on the symptomatic side in the 56 cases assessed by EJ and AH with Kappa. In Kaplan–Meier and Cox regression assessments, cases were censored at revascularization, loss to follow-up, death and 2 or 28 days after presenting event. p < 0.05 was pre-specified as statistically significant. IBM SPSS 28.0 were used in the analyses.

## Results

Of 402 patients with ≥ 50% stenosis or occlusion during the study period, we excluded 84 before detailed stenosis assessment (54 no consent, 12 major presenting stroke, 9 severe comorbidity, 7 severe kidney failure, 2 contrast allergy), and 200 without near-occlusion. Of 118 included symptomatic near-occlusions, 26 had full collapse. Full collapse was assessed the same by both observers in all 56 cases (kappa 1.0), none of the excluded cases (without near-occlusion) were mistaken for full collapse by the measurement criteria by either observer. Baseline comparisons are presented in data supplement.

**Fig. 2:**
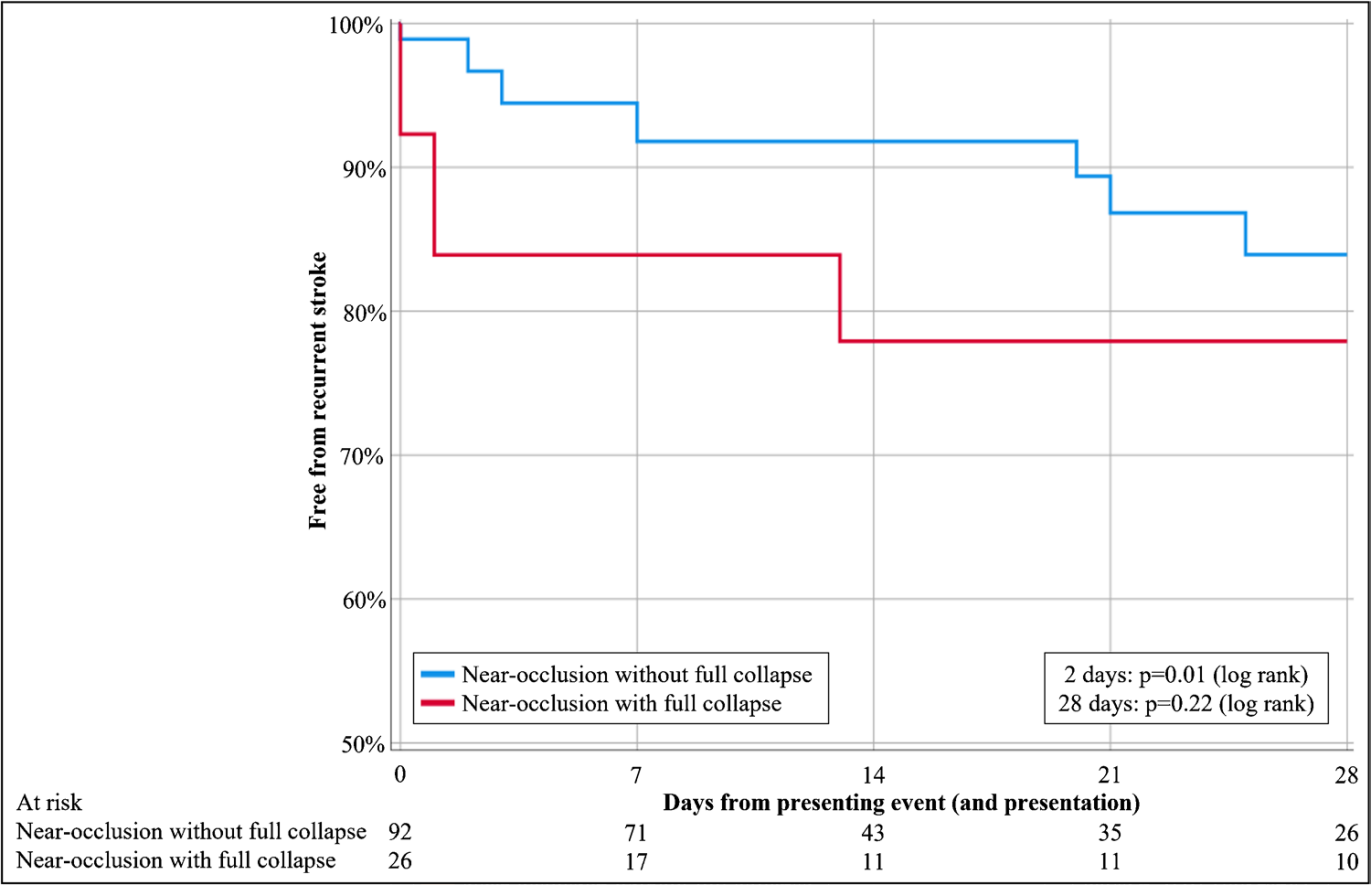
Kaplan Meier assessment of risk of recurrent stroke. Censoring for CEA/stenting, loss to follow-up or death

15 participants suffered a recurrent stroke within 28 days of the presenting event. At 2 days, the risk of recurrent stroke was 16% (95%CI 2–35%) in those with full collapse and 3% (95%CI 0–7%) in those without full collapse, p = 0.01 (log rank test). At 28 days, these risks were 22% (95%CI 4–40%) and 16% (95%CI 6–26%), p = 0.22 (log rank test, Fig. 2). We assessed if another threshold could improve the prognostic discrimination, but no relevant improvement was possible. None of the factors associated with full collapse were associated with risk of stroke recurrence: Sex p = 0–31 (log rank), low-density lipoprotein p = 0.94 (Cox regression) and contralateral stenosis p = 0.78 (log rank).

## Discussion

The main findings of this study were the high risk of early recurrent stroke (especially within 2 days of presenting event) in near-occlusion with full collapse defined as ≤ 2.0 mm and/or ICA ratio ≤ 0.42 and that this definition was very reliable between observers.

We found a high risk of recurrent stroke among near-occlusion with full collapse (16% at 2 days and 22% at 28 days) which is similar to the previous derivation analysis of the measurement suggested measurement-based definition of full collapse and hence validate this definition [8]. Also, even though 2 observers used different measurement approaches, the reliability was perfect in this sample. We do not expect perfect agreement in larger samples, but it is reasonable that the reliability will at least be good enough for clinical use. Hence, this definition can be used in future studies of near-occlusion with full collapse, including treatment trials.

Given the very high 2-day risk, treatment is very relevant to consider and must be hyperacute. However, we have found no study assessing the risks with such early treatment by any method.

CEA in subacute stage has technical feasibility issues [6] and might cause hyperperfusion intracerebral or subarachnoid hemorrhage [10]. Thus, any treatment attempts should be done within controlled trials. If so, the possibility of artery closure [10] might also be considered.

We found no clear risk difference between the groups at 28 days, albeit at 2 days. However, the aim of this study was to assess if the stroke risk was high in near-occlusion with full collapse defined by measurement definition, not to show that the risk is also higher than the control group. Near-occlusion without full collapse has had varying prognostic findings [3, 5–7], but revascularization might be warranted, further research is warranted.

Study limitations included smaller than expected sample of near-occlusion with full collapse compared to previous studies [6, 7], possibly due to cases mistaken for occlusion were not referred. This led to wide confidence intervals and precluded possibility of multivariable analyses (too few outcomes). However, as no factor was associated with both outcome and full collapse status, the risk of confounding seems low, similar to as in previous studies [5–7]. While within guidelines [4], it is possible that more aggressive medical therapy would result in fewer recurrent strokes.

In summary, near-occlusion with full collapse should be defined as distal ICA ≤ 2.0 mm and/or ICA ratio ≤ 0.42 as this definition defines a group with high risk of early stroke recurrence (especially within 2 days of presenting event) and is reliable between observers.

### Supplementary Information

Below is the link to the electronic supplementary material.

Supplementary file1 (DOCX 19 KB)

## Data Availability

The data produced in this study is available for additional analyses upon reasonable request, please contact corresponding author.
