# Supplementary material for: High risk of early recurrent stroke in patients with near-occlusion with full collapse of the internal carotid artery
Source: Neuroradiology. 2024 Jan 9;66(3):349–52. doi: 10.1007/s00234-024-03283-5 (PMC10859336; doi:10.1007/s00234-024-03283-5)
Supplement: Supplementary file 1 — Supplementary file1 (DOCX 19 KB) [file 234_2024_3283_MOESM1_ESM.docx]

|  | Near-occlusion without full collapse n=92 | Near-occlusion with full collapse n=26 | p^a^ |
| --- | --- | --- | --- |
| Age mean (SD) | 74 (7) | 73 (9) | 0.63 |
| Women n (%) | 21 (23) | 14 (54) | 0.004 |
| Previous myocardial infarction n (%) | 18 (20) | 6 (23) | 0.78 |
| Current angina n (%) | 11 (12) | 4 (15) | 0.74 |
| Current heart failure n (%) | 2 (2) | 1 (4) | 1.0 |
| Current symptomatic peripheral artery disease n (%) | 6 (7) | 1 (4) | 0.70 |
| Previous arterial revascularization^b^ n (%) | 21 (23) | 6 (23) | 1.0 |
| Previous stroke^c^ n (%) | 6 (7) | 3 (12) | 0.41 |
| Atrial fibrillation n (%) | 8 (9) | 4 (15) | 0.46 |
| Diabetes n (%) | 20 (21) | 8 (31) | 0.43 |
| Current smoker n (%) | 16 (17) | 7 (27) | 0.40 |
| Hypertension^d^ n (%) | 76 (83) | 22 (92) | 0.36 |
| Total cholesterol^b^ mmol/l mean (SD) | 4.2 (1.1) | 4.7 (1.3) | 0.11 |
| LDL cholesterol^b^ mmol/l mean (SD) | 2.2 (1.0) | 2.6 (1.2) | 0.06 |
| HDL cholesterol^b^ mmol/l mean (SD) | 1.2 (0.3) | 1.2 (0.3) | 0.95 |
| Referred n (%) | 78 (85) | 19 (73) | 0.24 |
| Presenting event: Amaurosis Fugax or RAO n (%) | 35 (38) | 5 (19) | 0.10 |
| Presenting event: TIA or stroke n (%) | 57 (62) | 21 (81) |  |
| Sought health care on the day of presenting event n (%) | 56 (61) | 19 (73) | 0.36 |
| Ipsilateral ischemic event <14 days before presenting event n (%) | 29 (32) | 6 (23) | 0.47 |
| Days between presenting event and CTA median (IQR) | 2 (0-12) | 0 (0-3) | 0.02 |
| CTA: Stenosis diameter^e^ median (IQR) | 0.5 (0.5-0.9) | 0.2 (0.2-0.5) | <0.001 |
| CTA: Distal ICA diameter median (IQR) | 3.1 (2.7-3.5) | 0.5 (0.2-1.4) | <0.001 |
| CTA: ICA ratio^f^ median (IQR) | 0.71 (0.64-0.78) | 0.16 (0.04-0.35) | <0.001 |
| CTA: ECA ratio median (IQR) | 1.07 (1.00-1.20) | 0.19 (0.07-0.49) | <0.001 |
| Contralateral 50-100% stenosis n (%) | 16 (17) | 12 (46)^g^ | 0.004 |
| Underwent revascularization n (%) | 73 (79) | 12 (46)^g^ | 0.001 |
| Days between presenting event revascularization median (IQR) | 11 (7-22) | 9 (2-14) | 0.28 |
| Type of revascularization: CEA n (%) | 64 (88) | 5 (42) | <0.001 |
| Type of revascularization: CAS n (%) | 9 (12) | 7 (58) |  |
| Any stroke or death <30 days of revascularization n (%) | 2 (3) | 1 (8) | 1.0 |
| CAS: Carotid stenting. CEA: Carotid endarterectomy. CTA: Computed tomography angiography. HDL: High-density lipoprotein. IQR: Inter-quartile range LDL: Low-density lipoprotein. SD: Standard deviation. RAO: Retinal artery occlusion. TIA: Transient ischemic attack.  ^a^ 2-sided χ^2^-test for categorical, One-way-ANOVA for continuous data presented as mean, Kruskal-Wallis for continuous data presented as median.  ^b^ 1-2 missing values  ^c^ >6 months before presenting event  ^d^ >140/90 mmHg and/or use of blood pressure reducing medication  ^e^ 4 missing values due to too calcified stenosis (2 in each group)  ^f^ 3 missing values due to contralateral occlusion (all in without full collapse group).  ^g^ 12 cases with full collapse in both these analyses. This was only numerically similar, there was no association between contralateral stenosis and undergoing revascularization in the full collapse group (p=0.71). | | | |

Supplemental table. Baseline comparisons.
